# Envelope-Specific IgG3 and IgG1 Responses Are Associated with Clearance of Acute Hepatitis C Virus Infection

**DOI:** 10.3390/v12010075

**Published:** 2020-01-08

**Authors:** Melanie R. Walker, Auda A. Eltahla, Michael M. Mina, Hui Li, Andrew R. Lloyd, Rowena A. Bull

**Affiliations:** 1 Viral Immunology Systems Program, The Kirby Institute, Sydney, NSW 2052, Australia; melanie.walker@unsw.edu.au (M.R.W.); a.eltahla@unsw.edu.au (A.A.E.); m.mina@unsw.edu.au (M.M.M.); hui.li@unsw.edu.au (H.L.); a.lloyd@unsw.edu.au (A.R.L.); 2 School of Medical Sciences, Faculty of Medicine, The University of New South Wales, Sydney, NSW 2052, Australia

**Keywords:** hepatitis C virus (HCV), humoral immunity, antibody subclasses, antibody isotypes, envelope

## Abstract

Hepatitis C virus (HCV) can be cleared naturally in a subset of individuals. However, the asymptomatic nature of acute HCV infection makes the study of the early immune response and defining the correlates of protection challenging. Despite this, there is now strong evidence implicating the humoral immune response, specifically neutralising antibodies, in determining the clearance or chronicity outcomes of primary HCV infection. In general, immunoglobulin G (IgG) plays the major role in viral neutralisation. However, there are limited investigations of anti-HCV envelope protein 2 (E2) isotypes (IgM, IgG, IgA) and IgG subclasses (IgG1–4) in early HCV infection. In this study, using a rare cohort of 14 very recently HCV-infected individuals (4–45 days) with varying disease outcome (*n* = 7 clearers), the timing and potency of anti-HCV E2 isotypes and IgG subclasses were examined longitudinally, in relation to neutralising antibody activity. Clearance was associated with anti-E2 IgG, specifically IgG1 and IgG3, and appeared essential to prevent the emergence of new HCV variants and the chronic infection outcome. Interestingly, these IgG responses were accompanied by IgM antibodies and were associated with neutralising antibody activity in the subjects who cleared infection. These findings provide novel insights into the early humoral immune response characteristics associated with HCV disease outcome.

## 1. Introduction

Hepatitis C virus (HCV) is a major cause of chronic liver disease globally [1,2]. It is predominantly transmitted via blood-to-blood contact associated with injecting drug use (IDU), via sharing of injecting equipment [3,4]. Following acute infection, approximately 75% of people fail to clear the virus, resulting in chronic hepatitis [5,6,7]. While direct-acting antiviral (DAAs) treatments are decreasing the number of people living with HCV, control remains challenging globally due to the limitations in health infrastructure and high drug cost for treatment of the marginalised population affected, in addition to high rates of re-infection [8,9,10,11,12,13]. As HCV can be cleared naturally in a subset of individuals (~25%), and as subsequent re-infections are characterised by higher clearance rates (up to ~80%), it is reasonable to hypothesise that a vaccine could be designed to elicit effective immune responses that confer protection [14,15].

As early HCV infection is predominantly asymptomatic, it is difficult to characterise the initial immune response that contributes to clearance of HCV. Despite this, recent evidence suggests that an early neutralising antibody (nAb) response targeting envelope (E) glycoproteins E1 and E2, which are responsible for entry into the host cells, may contribute to clearance of the virus [16,17,18,19]. Immunoglobulin G (IgG) is thought to play a major role in viral neutralisation [20,21,22,23]; however, anti-HCV envelope isotypes and IgG subclasses in early primary HCV infection were studied longitudinally in only limited case series [24,25]. Furthermore, previous studies were limited with the majority of assays performed on samples from subjects with ultimate chronic infection (termed here chronic progressors), and examined reactivity against the core and non-structural (NS) proteins [26,27,28,29,30,31,32,33,34]. The most informative single study performed to date suggested that the majority of HCV-specific antibodies directed against NS, core, and envelope proteins were in the IgG1 and IgG3 subclasses, with limited IgG2 and IgG4 detected [23]. HCV-specific IgG1 and IgG3 antibodies were found in both acute and chronic stages of infection, including both clearers and chronic progressors [23]. Additionally, HCV-specific IgA and IgM antibodies were found in both the acute and chronic stages of HCV infection, but were not found to be associated with clearance [26,29,34].

Understanding these associations is important for development of antibody-based vaccines, as varied antibody subclasses and isotypes were found to be important in controlling different viral infections. IgM was shown to be critical for protection against West Nile virus and human immunodeficiency virus (HIV) mucosal transmission [35,36], where the pentameric structure of IgM is proposed to have high avidity resulting in a slow or absent off rate [35,37]. IgM was also shown to have neutralising properties against chikungunya and influenza viruses [36,38,39] and an important role in early immune activation via its Fc portion [40]. Furthermore, an early neutralising IgM response is induced by the smallpox (vaccinia) vaccine, one of the most successful vaccines to date [38,41].

With regard to IgG, in primary HIV infection, IgG3 antibody responses rather than IgG1 responses are linked to nAb activity and a drop in viral load [22,42], with a positive correlation with IgG3 and IgG1 response patterns, over IgG2, IgG4, and IgA [43,44,45]. Results from the RV144 HIV vaccine trial performed in Thailand noted that subjects who developed IgG3 antibodies against the Env V1V2 regions had a reduced risk of infection [46,47]. IgG3 is a more flexible subclass, and it is hypothesised that this feature allows the antibody better access to the virus, resulting in superior neutralisation [22,42]. Additionally, samples from the same vaccine trial showed a higher anti-HIV envelope variable region (Env V1V2) IgG:IgA ratio correlated with a decreased risk of infection [43]. It was proposed that IgA may outcompete and block Fc-dependent protective functions of IgG-bound antibodies, such as antibody-dependent cell-mediated cytotoxicity (ADCC) responses [48].

Given this backdrop, it is possible in HCV infection that specific isotypes and subclasses with either nAb or non-nAb activity (i.e., Fc-dependent activities) directed against E1E2 could facilitate viral clearance [21,22]. Better understanding of the details of humoral immune-mediated protection is essential for successful vaccine design. We and others showed that early nAb responses against HCV were associated with clearance of primary infection [16,17,19]. In this study, we extended upon these findings and examined the isotype and subclass utilisation and the timing and potency of these responses, in relation to nAb activity, in a rare cohort of very recently infected individuals. The findings indicate that IgG, specifically IgG1 and IgG3, responses appear essential to prevent emergence of new HCV variants and the chronic infection outcome.

## 2. Materials and Methods

### 2.1. Subjects and Samples

Samples from a prospective cohort of 590 HCV seronegative, high-risk individuals enrolled in the Hepatitis C Incidence and Transmission Study (HITS) in prisons (HITS-p) or in a sister cohort in the general community (HITS-c) collected between 2005 and 2015 were used in this study [49,50,51]. Inmates were enrolled in 34 correctional centres across New South Wales, Australia. Blood samples were collected every six months to screen for seroconversion and for HCV RNA positivity; upon incident infection, subjects were sampled frequently for 24 weeks until infection outcome was resolved and antiviral treatment offered, and then three to six monthly thereafter. Early incident cases (*n* = 14), designated when a time point was available that was antibody-negative and HCV RNA-positive, were selected for this analysis. The date of infection was estimated by subtracting the average pre-seroconversion window period, which was estimated at 51 days [52,53,54,55], from the midpoint between the last seronegative and first seropositive time points. Details of the study protocol are reported elsewhere [12,49,50,51].

### 2.2. Ethics Statement

Ethical approvals were obtained from New South Wales Department of Corrective Services (reference number 05/0884), the University of New South Wales (reference numbers 05094, 08081), and Human Research Ethics Committees of Justice Health, (reference numbers GEN 31/05 and G304/11), 6 December 2011, all located in Sydney, Australia. Written informed consent was obtained from the participants. All methods were performed in accordance with the ethical guidelines of the 1975 Declaration of Helsinki.

### 2.3. Virologic Assessments

All sera were tested for HCV antibodies using the Abbott ARCHITECT anti-HCV chemiluminescent microparticle immunoassay (Abbott Diagnostics, Chicago, IL, USA). HCV RNA detection was performed using either the VERSANT HCV RNA Qualitative Transcription Mediated Amplification (TMA) assay (Bayer Diagnostics, Emeryville, CA, USA; lower limit of detection: 3200 copies/mL) for samples collected prior to July 2008, or the COBAS AmpliPrep/COBAS TaqMan HCV assay (Roche, Basel, Switzerland; lower limit of detection 223 genome copies/mL) for samples from August 2008 onward [56].

### 2.4. Construction and Expression of Recombinant E2

Recombinant E2 (rE2) protein was derived from HCV H77 genotype 1a (GenBank accession number AF011751) and HCV UNK3a.13.6 genotype 3a (GenBank accession number AY894683). E2 regions corresponding to HCV H77 genotype 1a isolate poly-protein amino acid residues 384 to 661, introduced into pcDNA3.1 expression vector (Life Technologies, Carlsbad, CA, USA) with a C-terminal Avitag and six-histidine epitope tag (6-His) [57,58] and codon-optimised, were purchased from GeneArt (Thermo Fisher Scientific, Waltham, MA, USA).

Transient transfection of expression plasmids was performed using 293-F cells (FreeStyle 293 HEKs, Thermo Fisher Scientific, Waltham, MA, USA) using DNA-293fectin reagent (Life technologies, Carlsbad, CA, USA) as per the manufacturer’s instructions. Media was harvested at approximately 96 h post transfection for purification. This supernatant was then passed through a 1-mL HiTrap Chelating HP chromatography column (Amersham, Buckinghamshire, UK) charged with 0.1 M NiSO_4_·6H_2_O and washed with 10 mL of binding buffer. Proteins were eluted from the column by passing through 50 mM imidazole in binding buffer. Elution fractions were pooled, and native PAGE, SDS-PAGE, and Western blotting were performed with the HCV-specific monoclonal antibody, CBH4G, as well as the AviTag antibody. Additionally, an ELISA was performed with nine previously characterised HCV monoclonal antibodies (mAbs) to confirm the conformation of the recombinant E2 (rE2) protein. These mAbs included AR1B, AR2A, AR3A, AR4A, and AR5A, sourced from A/Prof. Mansun Law (Scripps, La Jolla, CA, USA) [59], CBH4G and CBH7 antibodies purified from hybridomas purchased from the American Type Culture Collection (ATCC; Manassas, VA, USA, PTA-4468 and PTA-4470), and HCV84.26 and HCV-1 generated by transient transfection of plasmids provided by Prof. Heidi Drummer (Burnet Institute, Melbourne, Australia) as previously described [60].

### 2.5. Enzyme-Linked Immunoassays

Microtitre 96-well plates 96-well (Nunc Maxisorb, Thermo Fisher Scientific) were prepared with rE2 (H77 for genotype 1 and 2 infected subjects or UNK3a.13.6 for genotype 3 infected subjects) and incubated for one hour. Plates were washed three times with Tris-buffered saline with Tween (TBS-T) and then blocked for one hour with blocking buffer (5% non-fat dry milk in TBS-T). The bound rE2 was then incubated with plasma (heat inactivated by incubation at 56 °C for 30 min) at a final dilution of 1:10 for 1.5 h. The bound plasma was then incubated for one hour with anti-human IgG conjugated with horseradish peroxidase (HRP) (Jackson Immunoresearch, West Grove, PA, USA, 1:6000 for genotype 1a and 3a), anti-human IgA (α-chain-specific)–HRP (Sigma-Aldrich, St. Louis, MO, USA, 1:3000 for genotype 1a and 1:1500 for genotype 3a), anti-human IgM (μ-chain-specific)–HRP (Sigma-Aldrich, 1:3000 for genotype 1a and 1:1500 for genotype 3a), anti-human IgG1 Fc–HRP (SouthernBiotech, Birmingham, AL, USA, 1:6000 for genotype 1a and 1:1500 for genotype 3a), anti-human IgG2 Fc–HRP (SouthernBiotech, 1:6000 for genotype 1a and 1:1500 for genotype 3a), anti-human IgG3 hinge–HRP (SouthernBiotech, 1:6000 for genotype 1a and 3a), and anti-human IgG4 Fc–HRP (SouthernBiotech, 1:6000 for genotype 1a and 1:1500 for genotype 3a). The 3,3′,5,5′-tetramethylbenzidine (TMB) liquid substrate for ELISA (Sigma-Aldrich) was added to the plate and incubated for 15 min after absorbance was read at 450 nm. In order to define a true positive result, a cut-off value was calculated for each assay as the mean + 3 SD of signal/noise (S/N) values in plasma from 10 healthy donors; these were as follows: genotype 3a; 1.87 S/N for IgA, 1.39 S/N for IgG and 2.89 S/N for IgM, 1.54 S/N for IgG1, 1.54 S/N for IgG2, 1.33 S/N for IgG3, and 2.9 S/N for IgG4 and genotype 1a; 1.67 S/N for IgA, 1.68 S/N for IgG and 3.91 S/N for IgM, 1.19 S/N for IgG1, 1.17 S/N for IgG2, 1.74 S/N for IgG3, and 2.55 S/N for IgG4. Results were normalised and converted to S/N for analysis. The mAbs CBH-5 and CBH-7 were used as a positive control for IgG and IgG1, respectively. For the remaining subclasses and isotypes, serum samples that previously tested positive in comparable ELISAs were kindly supplied as positive controls (Dr Kim Wilson, National Reference Laboratory, Melbourne, Australia). Unless specified, all subclass and isotype assays were performed in intra-assay duplicates.

### 2.6. Statistical Analysis

All data analysis was performed and all graphs were created using GraphPad Prism Software (version 7.0, La Jolla, CA, USA) for Macintosh. Wilcoxon rank-sum tests, Kolmogorov–Smirnov tests and Mann–Whitney tests were used (where appropriate) to evaluate statistically significant differences between groups. Statistical significance was defined as a *p*-value less than 0.05.

For the analysis of temporal trends in relation to viraemia, the period during primary infection in which the HCV viral load consistently declined was designated here as at least two consecutive time points, immediately after the peak HCV viral load time point (occurring at <90 days post infection (DPI), as described previously [61,62]). To be included in this analysis, consecutive time points were required to decline by >0.076 log_10_ IU/mL when compared to the HCV viral load of the previous time point (corresponding with the intra-assay variability previously reported for the COBAS Taqman Assay [63,64]). All sequential time points that declined were included. The endpoint for inclusion in the analysis was an HCV RNA increase by >0.076 log_10_.

## 3. Results

### 3.1. Patient Description

Longitudinally collected samples from 14 newly viraemic, seronegative subjects were selected for this study. These 14 subjects were described extensively elsewhere [19]. In brief, the 14 subjects had a primary HCV infection with the most prevalent Australian strains, including genotypes 1a (*n* = 5), 1b (*n* = 3), 3a (*n* = 4), and 2b (*n* = 2) [13]. The estimated days post infection (DPI) of the initial infection time point ranged from four to 45 days (median 30). Of the 14 subjects, seven naturally cleared the primary infection, and seven developed chronic infection (Table 1).

### 3.2. The Timing and Magnitude of Anti-E2 IgA and IgM Are Not Associated with Clearance, Whereas an Early Anti-E2 IgG Response Is

Anti-E2 IgM, IgG, and IgA responses were examined in the longitudinally collected sera to determine which isotype responses were being generated and whether these responses were associated with clearance or chronic infection outcomes.

**Figure 1:**
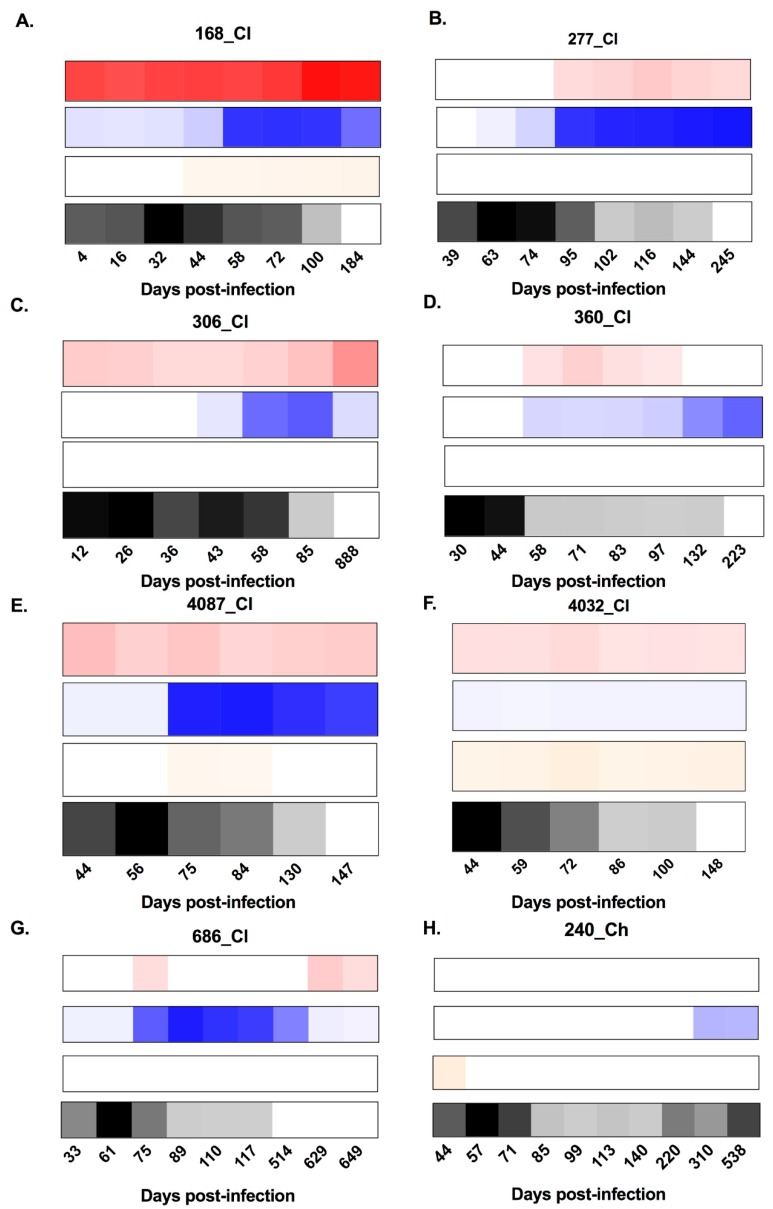

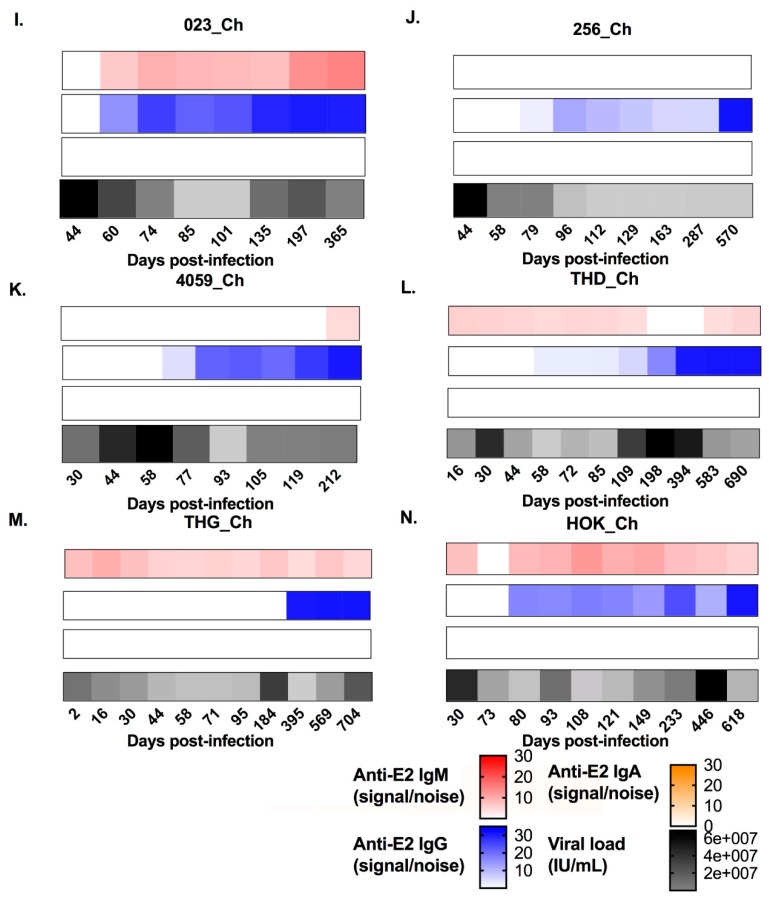
Heat maps representing the longitudinal patterns of anti-hepatitis C virus (HCV) envelope protein 2 (E2)-antibody isotype utilisation (signal/noise) for anti-E2 immunoglobulin M (IgM), IgG, and IgA responses, as well as HCV RNA levels (IU/mL). Panels **A**–**G** show subjects who cleared the infection. Panels **H**–**N** show subjects who developed chronic hepatitis. IgM is represented in red, IgG is represented in blue, IgA is represented in orange, and longitudinal HCV RNA levels (IU/mL) are represented in black (see key). Days post infection (DPI) are indicated below each heat map. All results were generated in duplicate from longitudinal serum samples collected for each subject.

**Figure 2:**
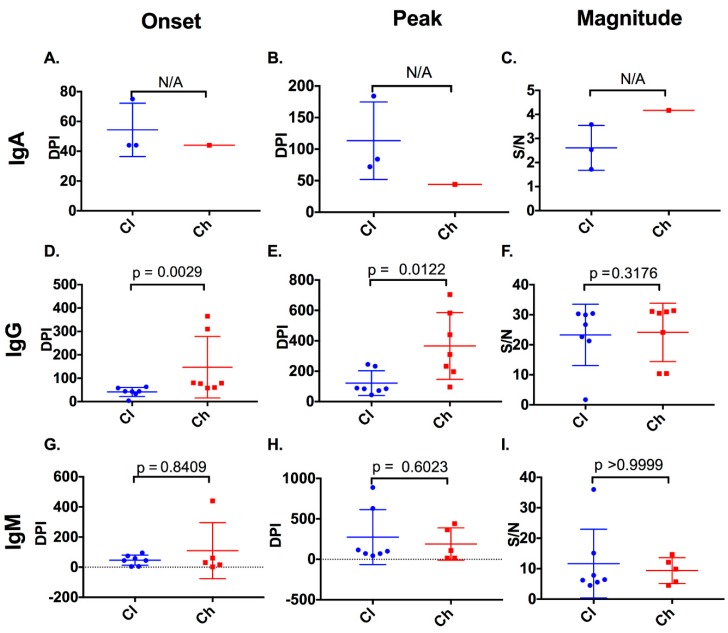
Timing and magnitude of anti-E2 IgA, IgG, and IgM responses in clearer and chronic progressor subjects. The timing (days post infection (DPI)) and magnitude (signal/noise (SN)) of anti-E2 IgA, IgG, and IgM responses were assessed in clearers and chronic progressors for the first detectable anti-E2 IgA (**A**), IgG (**D**), and IgM (**G**) responses, the timing of the peak anti-E2 IgA (**B**), IgG (**E**), and IgM (**H**) responses, and the magnitude of the peak anti-E2 IgA (**C**), IgG (**F**), and IgM (**I**) responses. Statistical significance was not applicable (N/A) for anti-E2 IgA responses, because of a lack of reactive samples in chronic progressors.

In this study, five of seven chronic progressors had detectable anti-E2 IgM reactivity, whereas all seven clearers had detectable anti-E2 IgM, at any one time during infection (Figure 1). In 10 of the 12 subjects with anti-E2 IgM, responses persisted and were relatively stable throughout infection. In the two remaining subjects, IgM responses were transient. The timing and magnitude of anti-E2 IgM responses were assessed in the clearers and chronic progressors with a reactive response to determine whether anti-E2 IgM was associated with disease outcome (Figure 2). No significant differences were observed in relation to the timing of the first detectable anti-E2 IgM response (Mann–Whitney; *p* = 0.8409, clearer median 44 DPI (range 4–95) versus chronic median 30 DPI (range 2–440)), timing of the peak response (DPI) (Mann–Whitney; *p* = 0.6023, clearer median 100 DPI (range 44–888) versus chronic median 108 DPI (range 16–440)), and magnitude of the peak anti-E2 IgM response (Mann–Whitney; *p* > 0.9999, clearer median 6.41 S/N, (range 4.5–36) versus chronic median 9.85 S/N, (range 4.56–14.65)).

Three of seven clearer subjects were found to have anti-E2 IgA responses, whereas only one of the seven chronic progressors was found to have anti-E2 IgA responses. In two of the subjects with anti-E2 IgA, responses were stable and persisted. In contrast, in the remaining two subjects, responses were transient (Figure 1). As only one chronic progressor had detectable anti-E2 IgA, the timing and magnitude of these responses were not compared statistically (Figure 2).

All seven clearers and all seven chronic progressors were found to have anti-E2 IgG responses. For the clearers, the anti-E2 IgG responses increased in magnitude up until ~100 DPI. Following this increase, levels were sustained for *n* = 3 subjects (277_Cl, 360_Cl, and 4032_Cl), whereas levels started to decrease concurrent with the loss of detectable virus in the plasma for the remaining *n* = 4 subjects (168_Cl, 306_Cl, 4087_Cl, and 686_Cl). This contrasted with the pattern observed in chronic progressors, where anti-E2 IgG levels increased over the course of infection, concordant with ongoing viraemia (Figure 1).

Clearers had an earlier anti-E2 IgG response (median of 44 DPI (range 5–74)) when compared to chronic progressors (median 80 DPI (range 58–365), Mann–Whitney; *p* = 0.0029). These dynamics were similar to those observed with nAb activity as recently published [19]. Additionally, clearers had an earlier peak anti-E2 IgG response (median 85 DPI (range 44–245)), when compared to chronic progressors (median of 310 DPI (range 96–704), Mann–Whitney; *p* = 0.0122) (Figure 2). However, no difference was observed in the magnitude of the response between clearers (median of 26.73 S/N (range 1.72–30.42)) and chronic progressors (median of 24.14 S/N (range 10.39–31.38), Mann–Whitney; *p* = 0.3176).

**Figure 3:**
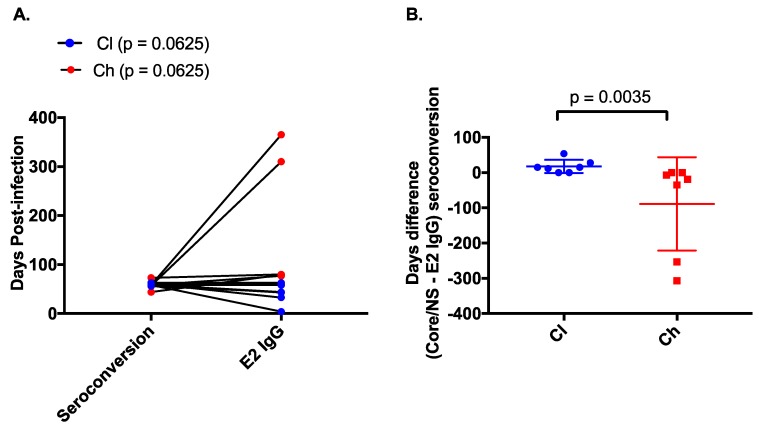
Comparison of E2-specific IgG binding and non-E2 specific binding in clearer and chronic progressor subjects. The timing (days post infection) of anti-E2 specific IgG as measured by ELISA was compared to the timing of core/non-structural protein (NS) seroconversion as measured by the commercial Abbott assay (**A**) for clearers (blue) and chronic progressors (red). The difference in days between the appearance of anti-E2 IgG and anti-core/NS was compared between clearers (blue) and chronic progressors (red) (**B**).

The timing of anti-E2 IgG responses was compared to the appearance of non-envelope antibody responses (i.e., anti-core, NS3 and NS5) measured using the Abbott ARCHITECT anti-HCV CIA assay. In clearers, anti-E2 IgG responses were detected before or in close proximity to development of responses against core and NS proteins (median of −15 days (range −54–0 days), Wilcoxon rank-sum test; *p* = 0.0625). In chronic progressors, anti-E2 IgG responses were often delayed until after the response to core and NS proteins (19 median days, range 0–307 days, Wilcoxon rank-sum test; *p* = 0.0625, Figure 3A). This time difference (seroconversion to core/NS (DPI)—appearance of anti-E2 IgG (DPI)) between clearers and chronic progressors was significant (*p* = 0.0035, Figure 3B).

### 3.3. An Early Anti-E2 IgG1 Response Is Associated with Clearance

The IgG subclass utilisation of the anti-E2 responses was examined to understand whether specific subclasses are utilised preferentially, as reported for HIV and other acute viral infections [21,65,66].

**Figure 4:**
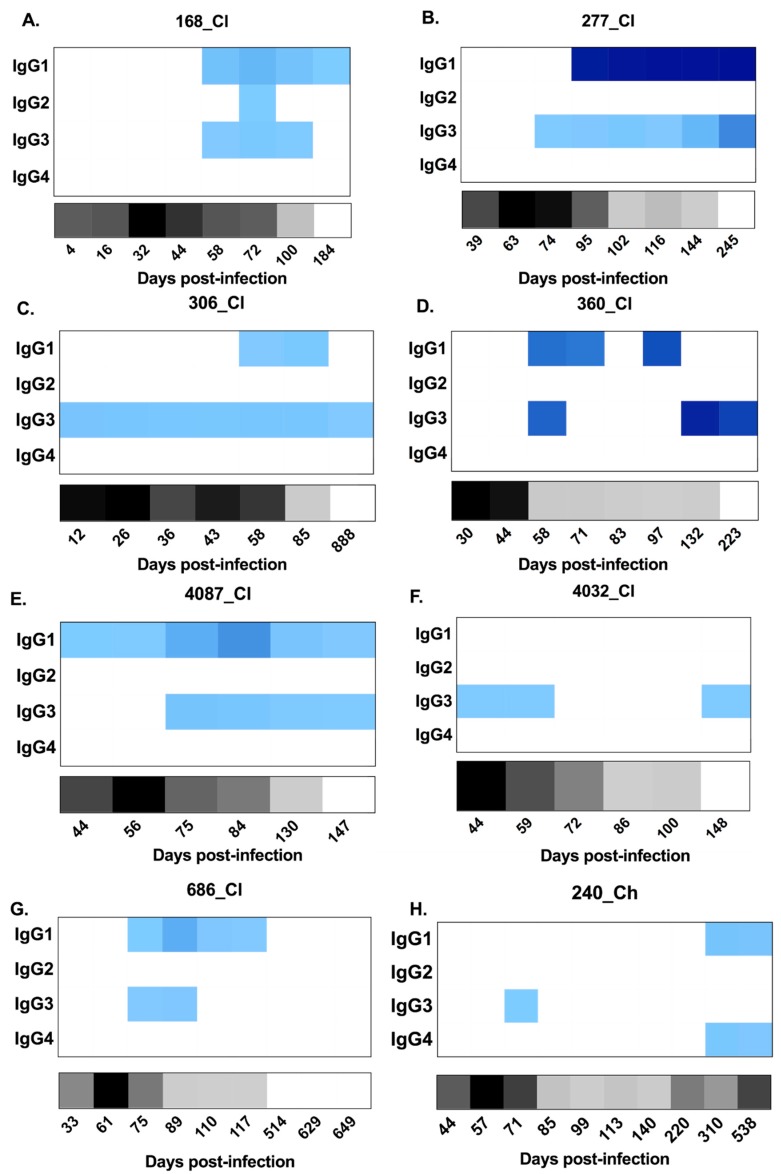

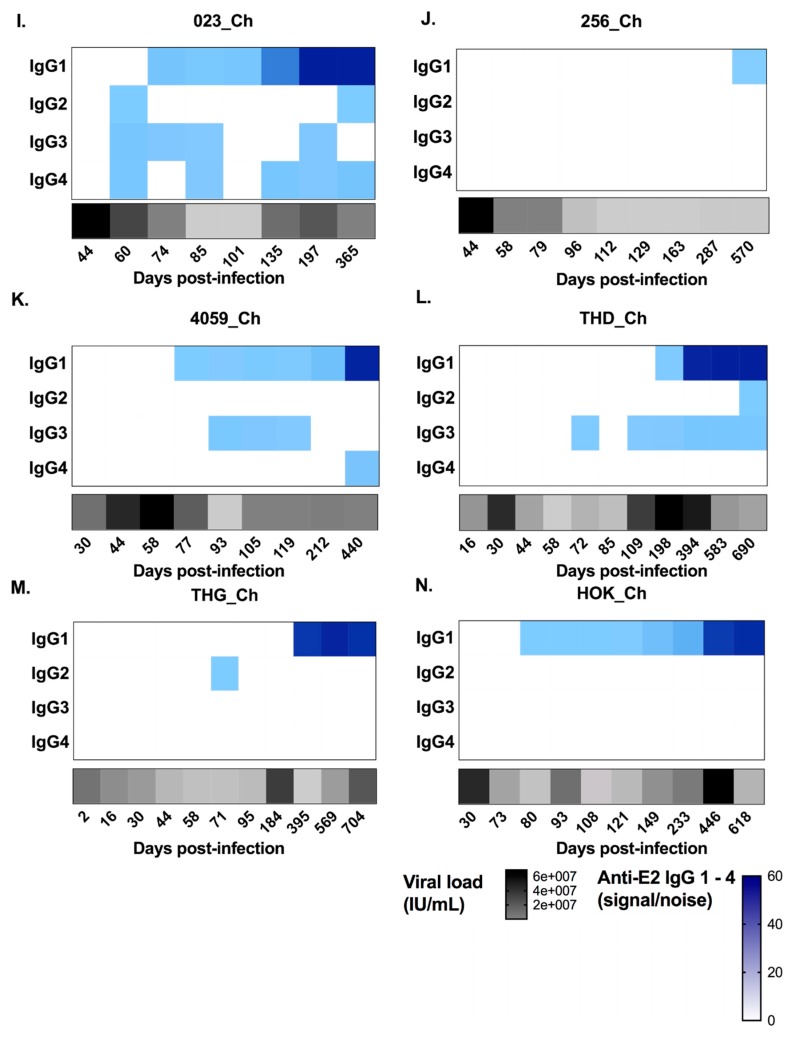
Heat maps representing longitudinal patterns of anti-HCV E2-antibody subclass utilisation (signal/noise) for IgG1–4 responses, as well as HCV RNA levels (IU/mL), in clearer and chronic progressor subjects. Panels **A**–**G** show subjects who cleared the infection. Panels **H**–**N** show subjects who developed chronic hepatitis. Anti-E2 IgG1–4 (signal/noise) is represented in blue with lower magnitudes in lighter blue and higher magnitudes in darker blue (see key). HCV RNA levels (IU/mL) are represented in grey/black (see key). Days post infection are indicated below each heat map. All results were generated from duplicate measures from all serum samples collected for each subject.

**Figure 5:**
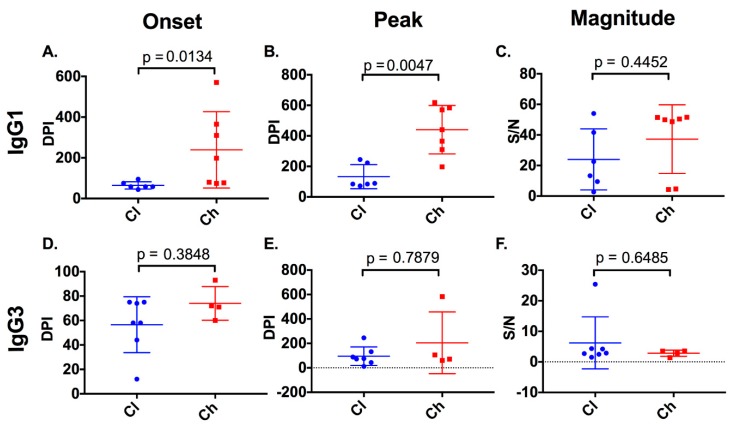
Timing and magnitude of anti-E2 IgG subclasses in clearer and chronic progressor subjects. The timing (days post infection (DPI)) and magnitude (signal/noise (SN)) of anti-E2 IgG1 and IgG3 responses were assessed in clearers and chronic progressors for the first detectable anti-E2 IgG1 (**A**) and IgG3 (**D**) responses, the timing of the peak anti-E2 IgG1 (**B**) and IgG3 (**E**) responses, and the magnitude of the peak anti-E2 IgG1 (**C**), IgG3 (**F**) responses.

Four of seven chronic progressors had detectable anti-E2 IgG3 responses, in comparison to all seven clearers (Figure 4). Responses were transient in eight of the eleven subjects lasting a mean of 66 days (SD 56). Analysis of the timing and magnitude (Figure 5) of these responses revealed no significant differences between clearers and chronic progressors for timing of the first anti-E2 IgG3 responses (*p* = 0.3848, clearer median 58 DPI (range 12–75) versus chronic median 71.5 DPI (range 60–93)), timing of the peak of anti-E2 IgG3 responses (Mann–Whitney; *p* = 0.7879, clearer median 74 DPI (range 12–245) versus chronic median 88 DPI (range 60–583)), and magnitude of the peak anti-E2 IgG3 responses (Mann–Whitney; *p* = 0.6485, clearer median 2.89 S/N (range 1.51–25.41) versus chronic median 3.185 S/N (range 1.39–3.58)).

With regard to the anti-E2 IgG1 response, all subjects, with the exception of 4032_Cl, had a response (Figure 4) which increased in magnitude in clearers until 100 DPI. Following this increase, IgG1 levels started to decline in all clearer subjects, except for 277_Cl where levels were sustained. In contrast, in all seven chronic progressors, the magnitude of the responses became stronger over time. When the timing of the first detectable anti-E2 IgG1 response for clearers and chronic progressors was compared, clearers were found to have significantly earlier responses (Mann–Whitney; *p* = 0.0134, clearer median 58 DPI (range 44–95) versus chronic median 198 DPI (range 74–570)) (Figure 5). Furthermore, clearers had significantly earlier peak anti-E2 IgG1 responses compared to chronic progressors (Mann–Whitney; *p* = 0.0047, clearer median 87 DPI (range 72–245) versus chronic median 440 DPI (range 197–618)). The peak anti-E2 IgG1 response was not significantly different (Mann–Whitney; *p* = 0.4452, clearer median 17.96 S/N (range 2.73–54.04) versus chronic median 49.88 S/N (range 4.27–51.57)).

Limited anti-E2 IgG2 was detected throughout infection, and the responses that were detected were transient and low in magnitude—only marginally above cut off (Figure 4). Similarly, anti-E2 IgG4 responses were not detected in any of the seven clearer subjects and in only three of the seven chronic progressors (Figure 4) in whom the responses were transient and low in magnitude.

### 3.4. The Initial Decline in HCV RNA Is Associated with Anti-E2 IgG1 and IgG3 Responses in Subjects Who Clear

**Figure 6:**
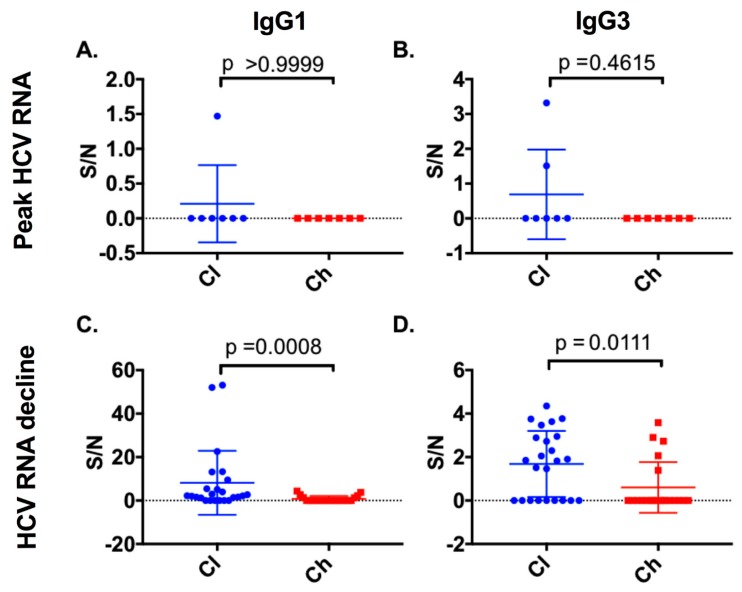
Anti-E2 IgG1 and IgG3 responses were examined at the peak HCV RNA time point and at the initial decline in viral load for both clearer and chronic progressor subjects. Anti-E2 IgG subclass responses at the peak HCV RNA time points were compared for anti-E2 IgG1 (**A**) or anti-E2 IgG3 (**B**). Additionally, time points where initial HCV RNA decline occurred were compared for clearers and chronic progressors for anti-E2 IgG1 (**C**) and anti-E2 IgG3 (**D**) responses.

At HCV RNA peak time points, all chronic progressors and the majority of clearer subjects were negative for IgG1 and IgG3. Therefore, no significant difference in IgG1 (Mann–Whitney; *p* > 0.9999) or IgG3 titres (Mann–Whitney; *p* = 0.4615) (Figure 6) was observed. The period referred to here as the HCV RNA decline (see Section 2 for definition) was evident in all seven clearers and seven chronic progressors between 16 and 130 DPI. At the HCV RNA decline (Table S1, Supplementary Materials) a potent IgG1 and IgG3 response was detected in all seven clearers. In contrast, the IgG1 and IgG3 responses in chronic progressors during this period were low or not detected (Mann–Whitney; *p* = 0.0008 and *p* = 0.0111 for anti-E2 IgG1 and IgG3, respectively) (Figure 6).

### 3.5. Co-Occurrence of IgM/IgG Occurs in Samples with nAb Activity in Subjects Who Clear

**Figure 7:**
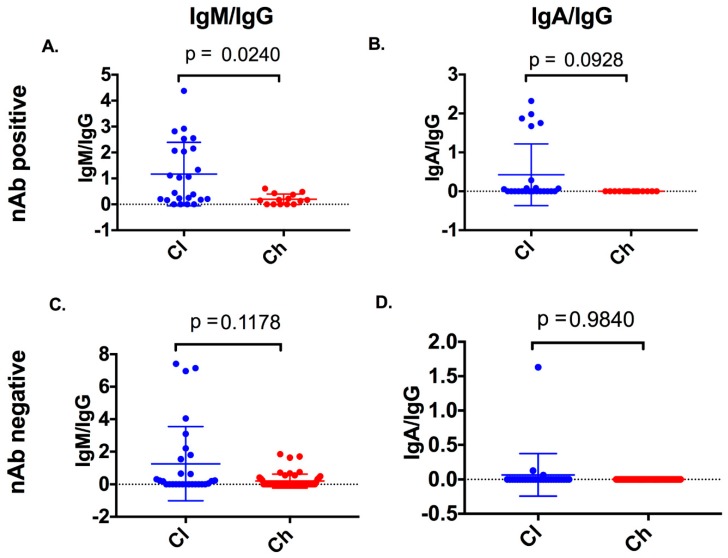
Anti-E2 isotype ratios were compared between clearers and chronic progressors at samples with neutralising antibody (nAb) activity and without nAb activity. Anti-E2 IgM/IgG and anti-E2 IgA/IgG was compared between clearers and chronic progressors in samples with nAb activity (nAb-positive, panels **A** and **B**) and without nAb activity (nAb-negative, panels **C** and **D**).

To understand isotype(s) which may correlate with nAb activity, the longitudinal nAb data generated on the same cohort and time points used in this study were analysed in the context of anti-E2 isotypes (Table S1, Supplementary Materials) [19]. In samples where nAb responses (50% inhibition of HCVpp at 1/40 dilution of serum) were present, IgG reactivity accompanied by either an anti-E2 IgM or an anti-E2 IgA response was observed. Therefore, the ratio of anti-E2 IgA/IgG and anti-E2 IgM/IgG was calculated for time points where nAb responses were detected, and for time points where nAb responses were not detected. Clearers were found to have significantly higher anti-E2 IgM/IgG (Kolmogorov–Smirnov; *p* = 0.0240) (Figure 7) at time points with nAb when compared to chronic progressors. No significant difference was observed between clearers and chronic progressors for anti-E2 IgM/IgG in time points where nAb responses were not detected (Kolmogorov–Smirnov; *p* = 0.1178). Furthermore, no significant difference was observed for anti-E2 IgA/IgG between clearers and chronic progressors in time points both with (Kolmogorov–Smirnov; *p* = 0.0928), and without nAbs detected (Kolmogorov–Smirnov; *p* = 0.9840).

## 4. Discussion

This study is the first to examine longitudinal anti-E2 isotype and subclass responses in very early primary HCV infection in subjects with clearance or chronic progression outcomes. Clearance was associated with earlier and more potent anti-E2 IgG, specifically IgG1 and IgG3, responses. Interestingly, these IgG responses were accompanied by IgM antibodies and correlated with nAb activity in the subjects who cleared infection. These results provide novel insights into the humoral immune response characteristics associated with disease outcome in early HCV infection.

It is generally believed in primary HCV infection that an anti-E2 IgG response is associated with nAb activity, which in turn is associated with viral clearance [20]. The results presented here show that chronic progressors develop anti-E2 IgG much later in infection than those that clear the virus. This delayed kinetics was most evident for IgG1 subclass responses, but anti-E2 IgG3 responses were also found in all clearer subjects but only a subset of chronic progressors. There were limited significant anti-E2 IgG2 or anti-E2 IgG4 responses found in either outcome group, consistent with previous reports [25,67,68,69].

The initial viral load decline was correlated with both the anti-E2 IgG1 and anti-E2 IgG3 in subjects that cleared infection and is consistent with our recent finding in these same subjects that HCV RNA decline was associated with nAb activity in those who cleared infection [19]. IgG1 and IgG3 were implicated as the major mediators of nAb across various viral infections including West Nile virus, dengue, and HIV [42,70,71,72]. These findings are also consistent with those of Chung et al., who observed in primary HIV infection that IgG3 antibodies likely co-operate with IgG1 to drive Fc-dependent functions, such as antibody-dependent cell-mediated cytotoxicity (ADCC) [66]. Thus, future vaccine efforts in HIV and HCV, as well as other viral infections, may need to induce both anti-E2 IgG1 and anti-E2 IgG3.

The delayed anti-E2 IgG responses observed in chronic progressors in this study highlights a key unresolved question in the immunopathogenesis of primary HCV infection—why only some subjects develop an early and strong anti-E2 IgG1 response. The potential contributors to these delayed kinetics include the immunogenicity of the E1E2 of the particular transmitted–founder (T/F) variants [73], the regulation of humoral response, and the characteristics of the B-cell repertoire [74]. The delayed induction of IgG1 in chronic progressors could indicate delayed class switching and somatic hypermutation of the antibody due to inadequate help by cluster of differentiation 4-positive (CD4^+^) follicular helper T cells, a concept that is supported by a previous study examining HCV-specific CD4^+^ T cell responses which showed that, in a subset of subjects from the same cohort, chronic progressors had higher CD4^+^ regulatory-to-effector ratios. This, along with two recent studies investigating follicular helper T cells and HCV disease progression [75,76], suggests that HCV disease outcome may be pre-determined by the host [77]. It would be interesting to examine the IgG1 responses in chronic progressors to determine if they require extensive maturation to appear, thus indicating that the pre-existing B-cell repertoire is a natural determinant of outcome, as observed in HIV [78].

This is the first study to show that early co-occurrence of IgM is associated with the neutralising activity in the plasma of subjects with a clearance outcome from primary infection. An early IgM response may be protective because of its pentameric structure which results in high-affinity antibodies which can neutralise viruses before the development of an IgG response [38]. IgM also plays an important role in the maturation of B-cell responses regulating B-cell tolerance as well as class switching to IgG and IgA [40,79]. Results of this study are in keeping with those of vaccination studies in HIV, which showed that subjects with a higher anti-envelope IgA:IgG ratio after vaccination were more likely to become infected, potentially indicating reduced protective immunity [43]. It may be noteworthy that the present study examined these virus-specific antibody responses in association with clearance, whereas the HIV vaccine study was investigating protection—that is, reduced potential for establishment of infection [43]. It is well recognised that IgG and IgA antibodies can have neutralisation- as well as Fc-dependent functions including ADCC [20,80].

Although the results reported here are both promising and informative for HCV pathogenesis and vaccine research, there are limitations in the current study. A key limitation was that the ELISA-based assays used here did not measure virion-bound antibody (that is, antibodies bound in immune complexes), only free antibodies in the plasma [81]. It was shown in HIV that the first detectable antibody responses are found in the form of immune complexes [82]. This may also be the case in primary HCV infection. Engagement in immune complex formation could explain why transient responses were observed in some subjects for both anti-E2 IgM and anti-E2 IgA. It should be noted, however, that several time points were examined including those which had very low levels of viraemia (i.e., available antigen) and transient responses were still observed. Another key area of further investigation to strengthen these data is the characterisation of class switching in HCV-specific B cells.

In conclusion, subjects able to mount an early anti-E2 IgG3 and anti-E2 IgG1 response, along with IgM responses, were more likely to achieve natural clearance of HCV. Thus, future vaccine design efforts may seek to promote such anti-E2 IgG1 and anti-E2 IgG3 responses, potentially along with IgM to foster nAb and potentially non-nAb activities.

## Figures and Tables

**Table 1:** Subject characteristics and time point analysis.

Subject ID ^a^	Age at Infection	Sex	Disease Outcome	GT ^b^	First Sampling Point (DPI ^c^)	Time to Clearance	Initial Viral Load	Ethnicity	HIV Co-Infection	HBV Co-Infection
168_Cl ^d^	24	M	Clearer	1b	4	142	10,989,916	Caucasian	N ^f^	Y ^g^
277_Cl	25	M	Clearer	3a	39	195	5,482,503	Caucasian	N	N
306_Cl	24	F	Clearer	1a/2b	5	487	8,462,679	Caucasian	N	Y
360_Cl	29	M	Clearer	3a	30	178	5,648,631	Caucasian	N	Y
4032_Cl	22	M	Clearer	3a	44	124	237,930	Aboriginal	N	N
4087_Cl	32	F	Clearer	1b	45	139	13,118,082	Caucasian	N	N
686_Cl	23	F	Clearer	1a	33	316	287,770	Caucasian	N	N
023_Ch ^e^	22	M	Chronic	1a	36		19,234,348	Caucasian	N	N
240_Ch	21	M	Chronic	3a	44		54,887	Caucasian	N	N
256_Ch	31	M	Chronic	1a	44		34,149,824	Aboriginal	N	N
4059_Ch	31	M	Chronic	1a/2b	30		3,676,682	Caucasian	N	N
HOK_Ch	26	F	Chronic	1b	30		733,849	Caucasian	N	N
THD_Ch	25	M	Chronic	1a	16		235,662	Caucasian	N	N
THG_Ch	28	M	Chronic	1a	2		140,200	Caucasian	N	N

^a^ Identifier; ^b^ genotype; ^c^ days post infection; ^d^ Cl—clearer outcome; ^e^ Ch—chronic outcome; ^f^ no; ^g^ yes. M—male; F—female.

## Supplementary Materials

The following are available online at https://www.mdpi.com/1999-4915/12/1/75/s1: Table S1: Initial HCV RNA decline in subjects with varied disease outcomes.
