# Supplementary material for: Envelope-Specific IgG3 and IgG1 Responses Are Associated with Clearance of Acute Hepatitis C Virus Infection
Source: Viruses. 2020 Jan 8;12(1):75. doi: 10.3390/v12010075 (PMC7019651; doi:10.3390/v12010075)
Supplement: Supplementary file 1 [file viruses-12-00075-s001.pdf]

Table S 1 Initial HCV RNA decline in subjects with varied disease outcomes

Subject	DPI[a]	HCV RNA levels log_{10} IU[b]/mL	HCV RNA change[c]	Time point selected	Anti-E2 IgG1 (S/N)[f]	Anti-E2 IgG3 (S/N)	[g] nAb activity
**023_Ch**	36	7.28					
	44	7.25	0.03	Peak[d]	0	0	N[h]
	60	6.91	0.34	Y[e]	0	3.58	N
	74	5.6	1.31	Y	4.34	2.9	Y
	85	3.51	2.09	Y	2.71	2.06	N
	101	2.6	0.91	Y	3.76	0	N
	135	6.45	-3.85		28.6	0	N
	197	6.77	-0.32		51.57	2.91	Y
	365	5.71	1.06		51.14	0	Y
**240_Ch**	44	4.74			0	0	N
	57	4.93	-0.19	Peak	0	0	N
	71	4.81	0.13	Y	0	1.39	N
	85	3.78	1.02	Y	0	0	N
	99	2.7	1.09	Y	0	0	N
	113	3.69	-0.99		0	0	N
	140	3.01	0.67		0	0	N
	220	4.65	-1.63		4.27	0	N
	538	4.79	-0.14		4.19	0	Y

Subject	DPI[a]	HCV RNA levels log10 IU[b]/mL	HCV RNA change[c]	Time point selected	Anti-E2 IgG1 (S/N)[f]	Anti-E2 IgG3 (S/N)	[g] nAb activity
**256_Ch**	44	7.53		Peak	0	0	N
	58	7.28	0.25	Y	0	0	N
	79	5.91	1.37	Y	0	0	N
	96	4.71	1.2	Y	0	0	N
	112	1.7	3.01	Y	0	0	N
	129	2.84	-1.14		0	0	N
	163	2.13	0.71		0	0	N
	287	4.17	-2.04		0	0	N
	570	2.86	1.31		4.62	0	Y
**4059_Ch**	30	6.57			0	0	N
	44	7.24	-0.68		0	0	N
	58	7.42	-0.18	Peak	0	0	N
	77	6.86	0.55	Y	1.27	0	N
	93	3.77	3.09	Y	2.26	2.73	N
	105	5.69	-1.92		2.33	2.84	N
	119	5.77	-0.08		1.93	2.24	N
	212	6.04	-0.27		6.09	0	Y
**HOK_Ch**	30	5.87		Peak	0	0	N

Subject	DPI[a]	HCV RNA levels log10 IU[b]/mL	HCV RNA change[c]	Time point selected	Anti-E2 IgG1 (S/N)[f]	Anti-E2 IgG3 (S/N)	[g] nAb activity
	73	5.24	0.62	Y	0	0	N
	80	4.65	0.6	Y	1.35	0	N
	93	5.61	-0.96		1.27	0	N
	108	4.4	1.21		1.38	0	N
	121	4.89	-0.49		1.5	0	N
	149	5.41	-0.51		6.5	0	N
	233	5.54	-0.14		11.98	0	N
	446	5.98	-0.44		45.54	0	Y
	618	5.01	0.97		48.67	0	Y
**THD_Ch**	16	5.37			0	0	N
	30	5.74	-0.37	Peak	0	0	N
	44	5.25	0.49	Y	0	0	N
	58	3.3	1.94	Y	0	0	N
	72	5.04	-1.73		0	1.76	N
	85	4.79	0.24		0	0	N
	109	5.7	-0.91		0	1.97	N
	198	5.83	-0.13		1.69	2.59	N
	394	5.77	0.06		49.81	3.48	Y
	583	5.35	0.42		51.4	3.53	Y
	690	5.26	0.09		51.52	3.23	Y

Subject	DPI[a]	HCV RNA levels log_{10} IU[b]/mL	HCV RNA change[c]	Time point selected	Anti-E2 IgG1 (S/N)[f]	Anti-E2 IgG3 (S/N)	[g] nAb activity
**THG_Ch**	2	5.15		Peak	0	0	N
	16	5	0.15	Y	0	0	N
	30	4.9	0.1	Y	0	0	N
	44	4.53	0.37	Y	0	0	N
	58	4.3	0.23	Y	0	0	N
	71	4.31	-0.01	Y	0	0	N
	95	4.45	-0.14		0	0	N
	184	5.35	-0.9		0	0	N
	395	2.8	2.55		46.16	0	Y
	569	4.88	-2.08		49.88	0	Y
	704	5.16	-0.28		48.16	0	Y
**168_Cl**	4	7.04			0	0	N
	16	7.1	-0.06		0	0	N
	32	7.59	-0.48	Peak	0	0	N
	44	7.36	0.23	Y	0	0	Y
	58	7.1	0.26	Y	5.44	2.05	Y
	72	7.02	0.08	Y	9.51	2.72	Y
	100	4.72	2.3	Y	5.1	1.85	Y
	184	0			1.36	0	N

Subject	DPI[a]	HCV RNA levels log10 IU[b]/mL	HCV RNA change[c]	Time point selected	Anti-E2 IgG1 (S/N)[f]	Anti-E2 IgG3 (S/N)	[g] nAb activity
**277_Cl**	39	6.74			0	0	N
	63	7.09	-0.36	Peak	0	0	N
	74	7.02	0.08	Y	0	1.46	N
	95	6.54	0.48	Y	52.04	2.95	Y
	102	4.02	2.52	Y	53.09	3.47	Y
	116	4.85	-0.82		53.83	2.61	Y
	144	2.92	1.93		53.57	9.3	Y
	245	0			54.02	25.4	N
**306_Cl**	12	7.04			0	4.2	N
	26	7.11	-0.07		0	3.58	N
	36	6.76	0.35		0	3.31	N
	43	6.98	-0.22	Peak	0	3.32	N
	58	6.87	0.12	Y	2.18	3.77	N
	85	3.64	3.23	Y	2.73	3.63	Y
	888	0			0	2.29	N
**360_Cl**	30	6.75		Peak	0	0	N
	44	6.66	0.09	Y	0	0	N
	58	4.15	2.51	Y	1.64	1.82	N

Subject	DPI[a]	HCV RNA levels log_{10} IU[b]/mL	HCV RNA change[c]	Time point selected	Anti-E2 IgG1 (S/N)[f]	Anti-E2 IgG3 (S/N)	[g] nAb activity
	71	4.2	-0.05	Y	1.54	0	Y
	83	3.03	1.18	Y	0	0	N
	97	1.18	1.85	Y	2.03	0	Y
	132	1.76	-0.58		11.29	2.49	Y
	223	0			41.66	2.19	Y
**686_Cl**	33	6.38	-6.38		0	0	N
	61	7.84	-1.46	Peak	0	0	N
	75	6.71	1.13	Y	1.24	2.3	N
	89	3.36	3.35	Y	13.33	2.89	N
	110	1.18	2.18	Y	2.98	0	Y
	117	1.18	0	Y	2.26	0	Y
	514	0			0	0	
	629	0			0	0	Y
	649	0			0	0	
**4032_Cl**	44	5.38		Peak	0	1.51	N
	59	5.21	0.16	Y	0	1.51	Y
	72	5.07	0.14	Y	0	0	Y
	86	1.18	3.9	Y	0	0	Y
	100	3.47	-2.29		0	0	Y

Subject	DPI[a]	HCV RNA levels log_{10} IU[b]/mL	HCV RNA change[c]	Time point selected	Anti-E2 IgG1 (S/N)[f]	Anti-E2 IgG3 (S/N)	[g] nAb activity
	148	0			0	1.44	Y
**4087_Cl**	44	7.12			1.27	0	N
	56	7.47	-0.35	Peak	1.47	0	Y
	75	6.81	0.66	Y	13.15	4.35	N
	84	6.29	0.52	Y	22.58	3.75	Y
	130	1.4	4.89	Y	3.98	1.9	N
	147	0			2.39	1.87	N

[a] Days post-infection, [b] International Units, [c] HCV RNA change when compared to previous time point, [d] Peak HCV RNA time points, [e] Yes, [f] Signal to noise, [g] Neutralising antibody, [h] No
